# Nickel-Doped Graphite and Fusible Alloy Bilayer Back Electrode for Vacuum-Free Perovskite Solar Cells

**DOI:** 10.1021/acsenergylett.3c00852

**Published:** 2023-06-07

**Authors:** Mengyuan Li, So Yeon Park, Jianxin Wang, Ding Zheng, Owen S. Wostoupal, Xudong Xiao, Zhenzhen Yang, Xun Li, Benjamin T. Diroll, Tobin J. Marks, Kai Zhu, Tao Xu

**Affiliations:** † Department of Chemistry and Biochemistry, Northern Illinois University, DeKalb, Illinois 60115, United States; ‡ Chemistry and Nanoscience Center, National Renewable Energy Laboratory, Golden, Colorado 80401, United States; § Department of Chemistry and the Materials Research Center, Northwestern University, Evanston, Illinois 60208, United States; ∥ Chemical Sciences and Engineering Division, Argonne National Laboratory, Lemont, Illinois 60439, United States; ⊥ Center for Nanoscale Materials, Argonne National Laboratory, Lemont, Illinois 60439, Illinois 60439, United States

## Abstract

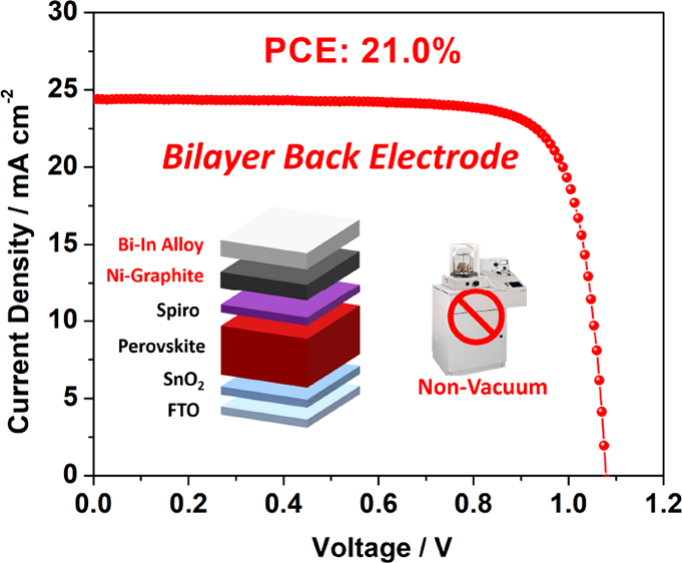

With the rapid development of perovskite solar cells (PSCs), lowering fabrication costs for PSCs has become a prominent challenge for commercialization. At present, gold is commonly used as the back metal electrode in state-of-the-art n-i-p structured PSCs due to its compatible work function, chemical inertness, and high conductivity. However, the high cost of gold and the expensive and time-consuming vacuum-based thin-film coating facilities may impede large-scale industrialization of PSCs. Here, we report a bilayer back electrode configuration consisting of an Ni-doped natural graphite layer with a fusible Bi-In alloy. This back electrode can be deposited in a vacuum-free approach and enables PSCs with a power conversion efficiency of 21.0%. These inexpensive materials and facile ambient fabrication techniques provide an appealing disruptive solution to low-cost PSC industrialization.

Organic–inorganic hybrid perovskite solar cells (PSCs), a promising solution-processed photovoltaic (PV) technology, have achieved a commercially appealing power conversion efficiency (PCE) of 25.7%, which is comparable to that of silicon-based PVs.^1^ Although the cost of processing the perovskite layer is low, other device layers involve high-cost materials and expensive fabrication equipment and facilities, which may hamper the large-scale PSC deployment. Specifically, the fabrication process for most state-of-the-art PSCs relies on a time- and energy-inefficient vacuum-coating process to deposit back electrodes such as gold (Au).^2^ Indeed, PSCs with record certified PCEs typically use an n-i-p configuration with gold as the back electrode.^3^ Because their work function (WF) is comparable to that of gold, carbon materials are regarded as the ideal low-cost substitution for gold.^4^ However, the low electrical conductivity of carbon materials^5^ can result in high series resistance, leading to unsatisfactory PV performance.

In recent years, vacuum-free coating techniques to fabricate carbon-based back electrodes have been explored.^6^ However, only a handful of reported PSCs with carbon-based back electrodes fabricated via a vacuum-free process achieved PCEs of over 18%. Previously, carbon electrodes were often fabricated by coating carbon paste directly on PSCs via a doctor-blade method, which produced PCEs of over 18%.^7−9^ Subsequently, a commercial carbon paste was used to prepare a self-adhesive macroporous carbon film via a solvent-exchange method; the carbon film was then coated on PSCs through a press transfer process, achieving a PCE of 19.2%.^9^ More recently, this method was applied to the optimized device, attaining a PCE of over 20%.^10^ An improved approach employed graphite paper laminated with a self-adhesive carbon film to lower the sheet resistance, yielding PSCs with PCEs of over 18% on small areas (0.1 cm^2^) and over 17% on large areas (1.0 cm^2^).^11,12^ Another study reported that a new type of PSC that must perform under permanent pressurization had a PCE reaching 18.6%.^13^ The PCE of these PSCs under permanent pressurization was further increased to over 21.6% using single-atom titanium (Ti)-doped reduced graphene oxide (rGO) coated on a fluorine-doped tin oxide (FTO) electrode as the back electrode, where the Ti dopant is to adjust and align the WF of rGO with the Fermi level of spiro-OMeTAD.^14^ Replacing rGO with other high-cost carbon materials, such as multiwalled carbon nanotubes (MWCNTs) and single-walled carbon nanotubes (SWCNTs), boosted PCEs to 22.2% and 21.4%, respectively, but permanent pressurization is still required.^15^ Defective multiwalled carbon nanotubes (D-MWCNTs) were another efficient carbon material that enabled a PCE of over 22% when devices were under permanent pressurization.^16^ Recently, a new type of PSC, which uses hot-pressed copper–nickel–graphene as the back electrode and is prepared by vacuum-based physical vapor deposition (PVD) and chemical vapor deposition (CVD), was reported with a PCE of over 24%.^17^

In contrast to the tight and compact coverage created by vacuum coating, both flexible adhesive carbon films and pressurized carbon electrodes inevitably incur voids at the interface with the underlying layer, leading to a loss of current pathways. In addition, pressurized carbon electrodes require an additional apparatus in the final device package to maintain homogeneous permanent pressure over the entire device lifetime and to ensure uniform interfacial contact. This is a challenging operation and maintenance requirement in large-area devices. Thus, a technology that inherits seamless interfacial contact imparted by vacuum coating while circumventing the costly vacuum process and the impractical on-device pressurization is a pressing need for PSC industrialization.

Herein, we report an innovative vacuum-free with a low-cost-materials-based bilayer back electrode configuration suitable for n-i-p structured PSCs without pressurization. This bilayer electrode consists of two sequentially coated layers to impart the charge extraction and charge transport to two different layers with respectively pertinent features: a nickel-doped natural graphite layer (Ni-G) with a proper WF for interfacial charge extraction, followed by a compact low-temperature fusible bismuth-indium (Bi-In) alloy layer for charge transport. Due to the atomically flat 2D structures and van der Waals bonding between graphene layers, the graphite layer can be seamlessly overlaid onto the hole-transporting layer (HTL) by simple rubbing. Furthermore, doping nickel particles into the graphite remarkably suppresses the alloy ingress into the graphite layer and optimizes the WF alignment with the HTL Fermi level. Note also that the fusible alloy layer melts at 110 °C and can then be painted onto the graphite layer, forming a seamless layer under an ambient atmosphere. This fusible upper alloy layer reduces the serial resistance of the entire back electrode. The result is a FAPbI_3_-based PSC device with a PCE of 21.0%. This bilayer back electrode configuration therefore offers a practical approach to low-cost, vacuum-free PSC fabrication without pressurization.

Figure S1 shows the synthetic route of Ni-doped graphite and images of Ni microparticles, natural graphite flakes, and the resulting Ni-doped graphite. These powders can be dispersed in ethanol under ultrasonication (Figure S2), forming a stable suspension. This method of coating a undoped graphite (G) or Ni-doped graphite (Ni-G) layer is similar to mechanical polishing. Specifically, a foam swab is dip-stained with G or Ni-G, which is then coated on the spiro-OMeTAD layer by rubbing the swab (Figure 1a). The G or Ni-G adheres to the spiro-OMeTAD layer due to the mechanical lubricity of graphite materials. A fusible Bi-In alloy consisting of 50:50 wt % bismuth:indium was prepared with a melting point of 89.5 °C, according to the Bi-In alloy phase diagram (Figure S3). This alloy is applied to the graphite layer by using a paint brush fully stained with molten alloy (Figure 1b), and the device size is defined by Kapton tape, as shown in Figure S4. The alloy layer solidifies when the PSC device is taken away from the hot plate.

**Figure 1:**
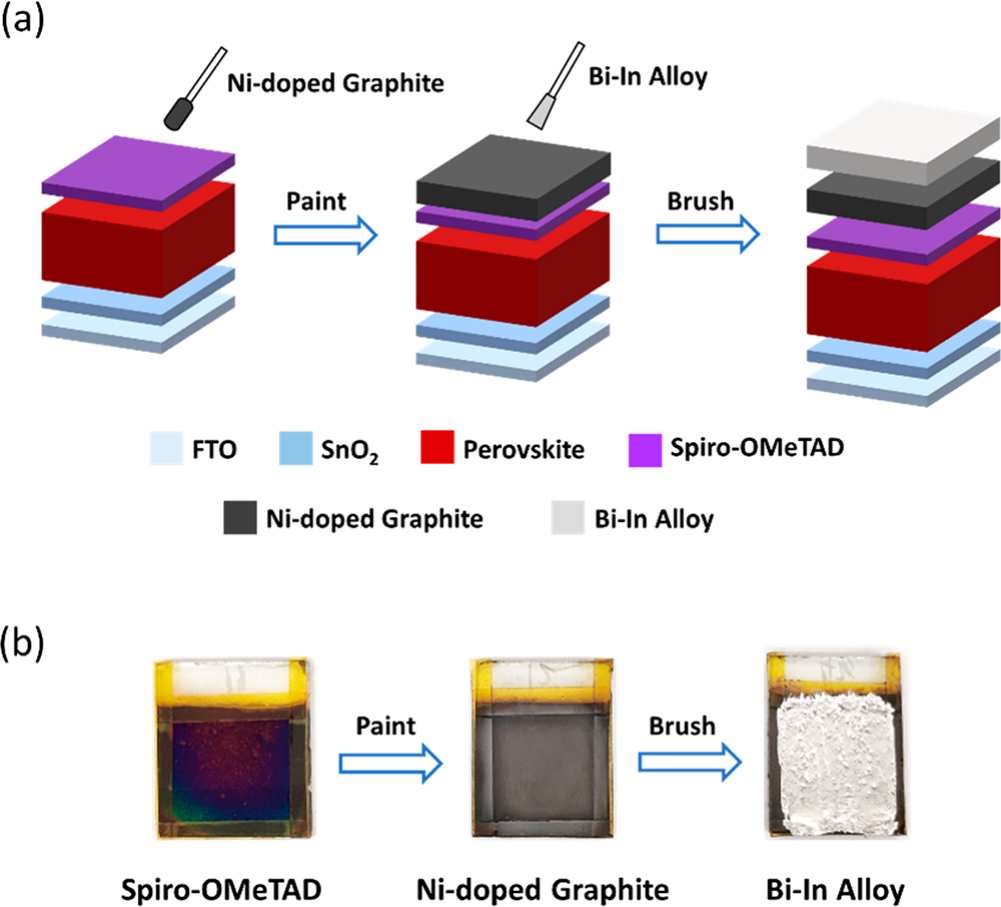
(a) Fabrication of a graphite-alloy bilayer back electrode on a PSC device. (b) Top-view image of spiro-OMeTAD, Ni-doped graphite, and Bi-In alloy.

FA_0.85_MA_0.1_Cs_0.05_Pb(I_0.9_Br_0.1_)_3_-based PSCs were prepared as a test bed to evaluate the PV performance of various back electrodes. For comparison, pure alloy and pure undoped graphite were also used as back electrodes and provided PCEs of 0.9% and 2.6%, respectively (Figure S5a,b). A similar PSC using Au as the back electrode achieved a PCE of 20.1% (Figure S5c). Figure 2a,b shows the current density–voltage (*J–V*) curves of PSCs using undoped graphite (G)/Bi-In alloy and 10 wt % Ni-doped graphite (10Ni-G)/Bi-In alloy bilayers as back electrodes, respectively. In stark contrast to the PSCs using pure alloy and pure graphite as the back electrodes, the PSCs using G/Bi-In and 10Ni-G/Bi-In bilayers as the back electrodes exhibit superior PCEs of 11.0% and 18.3%, respectively. With a higher doping of Ni, PSC based on 20 wt % Ni-doped graphite (20Ni-G)/Bi-In alloy bilayers exhibited a lower PCE of 14.8% (Figure S5d) compared with 10Ni-G.

**Figure 2:**
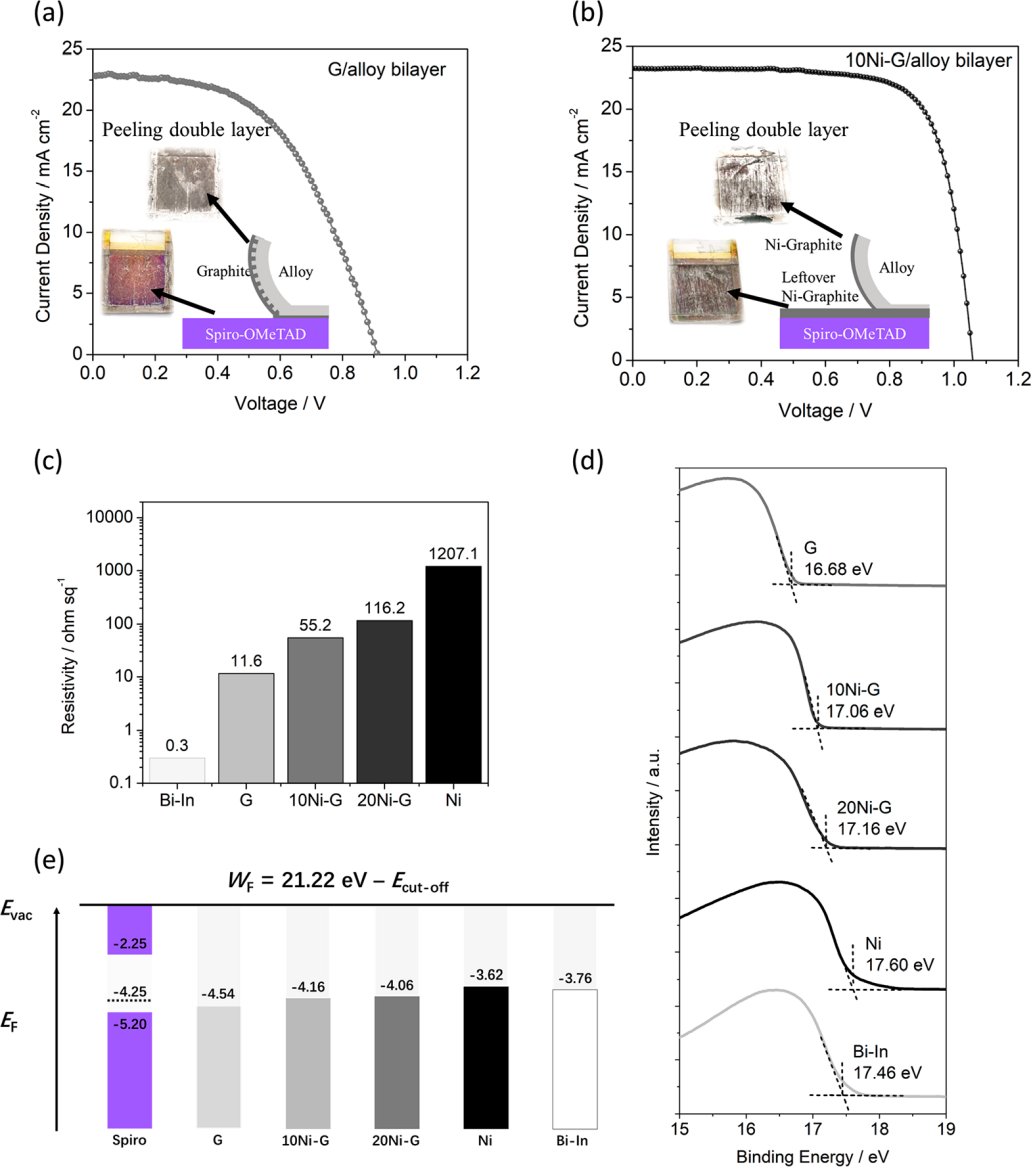
(a) *J–V* curves of a PSC device based on G/alloy bilayer back electrode. (b) *J–V* curve of a PSC device based on a 10Ni-G/alloy bilayer back electrode. (c) Square resistivities of Bi-In alloy, G, 10Ni-G, 20Ni-G, and Ni. (d) UPS spectra of Bi-In alloy, G, 10Ni-G, 20Ni-G, and Ni. (e) Energy diagram of spiro-OMeTAD,^13^ Bi-In alloy, G, 10Ni-G, 20Ni-G, and Ni.

To garner insight from the PCE data, four-probe measurements were carried out to examine the conductivity of these back electrode materials. As Figure 2c depicts, the sheet resistances of the Bi-In alloy and undoped graphite are 0.3 Ω sq^–1^ and 11.6 Ω sq^–1^, respectively. The PSC devices based on undoped graphite exhibited a low PCE of 2.6%, partly due to the higher resistivity of undoped graphite than of the Bi-In alloy. Furthermore, UV photoelectron spectroscopy (UPS) was conducted to obtain the WFs of these materials (Figure 2d). Figure 2e compares the Fermi energy level of spiro-OMeTAD (−4.25 eV) with the WFs of G, 10Ni-G, 20Ni-G, Ni microparticles, and the Bi-In alloy, which are −4.54, −4.16, −4.06, −3.62, and −3.76 eV, respectively. It is thus clear that although the Bi-In alloy has greater electrical conductivity than undoped graphite, the mismatch between the WF of the Bi-In alloy and the Fermi level of the HTL (spiro-OMeTAD) are the cause of the poor PCE.

It is therefore evident that simultaneously obtaining a proper WF and lower serial resistance is key to designing the back electrode for high PCEs, as exemplified by the present bilayer electrodes made from graphite/Bi-In (G/Bi-In) and Ni-G/Bi-In. It is still desirable to further fine-tune the WF of graphite, because the *V*_oc_ of the PSC using the G/Bi-In bilayer electrode is still only 0.91 V. This is likely reflecting the leaking through the graphite layer by the Bi-In alloy that contacts the spiro-OMeTAD, as shown in the inset of Figure 2a. The entire graphite layer was percolated by the alloy such that the whole graphite layer can be peeled off, leaving nearly no graphite remaining on the spiro-OMeTAD surface. Energy-dispersive X-ray (EDX) analysis of the counter surface of the Bi-In layer (Figure S6a) shows that 72 wt % of G/Bi-In is Bi-In alloy. In contrast, Figure S6b shows 31 wt % Bi-In alloy for Ni-G/Bi-In, demonstrating doping Ni can effectively inhibit the alloy ingression into graphite.

As Figure S7 shows, Ni is impermeable to Bi-In alloy so that the doping of Ni in graphite can greatly enhance the resistance of the graphite layer against the alloy ingression. In addition, this dopant must contribute to the WF alignment of the back electrode with spiro-OMeTAD Fermi level (−4.25 eV).^14^ Bulk crystalline Ni has a WF of 5.04 eV,^18^ and the WF of G is 4.54 eV. Thus, we adopt an amorphous Ni micropowder (evidenced by X-ray diffraction in Figure S8) with reduced WF^19^ as a dopant to optimize the overall WF of Ni-G in alignment with the Fermi level of spiro-OMeTAD (Figure 2e). By balancing the resistivity and the WF, the optimized doping concentration of Ni microparticles in graphite is determined to be 10 wt %.

The morphology of nickel powder and natural graphite was studied via scanning electron microscopy (SEM), showing a mixture of well-defined dots in the sub-micrometer range and flakes with sizes of a few tens of micrometers (Figure S9). The uniformity of the Ni doping was assayed by an EDX analysis as illustrated in Figure S10, which shows a homogeneously dispersed Ni signal in graphite. An X-ray photoelectron spectroscopy (XPS) analysis shows that the Ni particles have a binding energy of 852.2 eV, in agreement with metallic Ni after removing a thin surface oxide layer by Ar^+^ milling (Figure S11). As a result, the alloy does not wet through the Ni-microparticle-doped graphite layer, leaving only graphite powders on the spiro-OMeTAD surface upon peeling off the alloy layer as shown in the inset of Figure 2b. To investigate the interfacial charge transfer behavior between spiro-OMeTAD and graphite layer, steady-state photoluminescence (SSPL) and time-resolved photoluminescence (TRPL) measurements were conducted. Compared with spiro/G interface, spiro/10Ni-G exhibited higher luminescence quenching efficiency in a SSPL test (Figure S12a) and shorter luminescence lifetime in a TRPL test (Figure S12b), which synergically result in a more effective charge transfer at the spiro/10Ni-G interface. The suitable WFs of 10Ni-G and p-type NiO surface layer of Ni particles contribute to hole extraction from spiro-OMeTAD.^20^Table 1 summarizes all the PV performance data of PSCs with G/alloy and 10Ni-G/alloy bilayer back electrodes. The PSC device using 10Ni-G/alloy bilayer as the back electrode exhibited a better performance, with a short-circuit current density (*J*_sc_) of 23.3 mA cm^–2^, an open-circuit voltage (*V*_oc_) of 1.06 V, a fill factor (FF) of 0.74, and a PCE of 18.3%.

**Table 1:** Photovoltaic Parameters of PSC Devices Fabricated with Back Electrodes with Structures of G/Alloy Bilayer and 10Ni-G/Alloy

back electrode	*J*_sc_ (mA cm^–2^)	*V*_oc_ (V)	FF	PCE (%)
G/alloy bilayer	22.9	0.91	0.53	11.0
10Ni-G/alloy bilayer	23.3	1.06	0.74	18.3

To explore the potential of 10Ni-G/alloy bilayer back electrodes in high-performance PSCs, we further prepared PSCs using a FAPbI_3_-based perovskite absorber. Figure 3a shows the 10Ni-G/alloy-based PSC with an active area of 0.12 cm^2^ exhibiting a *J*_sc_ of 24.4 mA cm^–2^, a *V*_oc_ of 1.08 V, a FF of 0.79, and a PCE of 21.0%. Its Au-based counterpart PSC showed a PCE of 22.8%. Figure 3b shows the corresponding incident photon to current efficiency (IPCE) spectra and integrated current density of PSCs with a 10Ni-G/alloy bilayer and vacuum-evaporated Au layer as the back electrode, respectively. Both the IPCE spectra and integrated current density are in good agreement with their respective *J*_sc_ values. Statistical boxplots of PV parameters (Figure S13) demonstrate the comparable reproducibility of 10Ni-G/alloy bilayer electrode with Au. In addition, the fact that the sheet resistance of our Bi-In alloy layer (0.3 Ω/sq) is much less than that of FTO (∼10 Ω/sq) allows us to achieve large device sizes with a compatible PCE. The much lower sheet resistance of Bi-In alloy than that of FTO assures that our bilayer electrode is not the bottleneck in the presence of FTO in devices with a large active area. Thus, we also prepared 10Ni-G/alloy-based PSC devices with a large active area of 1 cm^2^; these devices exhibited a PCE of 18.7% (Figure 3c). The detailed PV parameters of these devices are summarized in Table 2.

**Figure 3:**
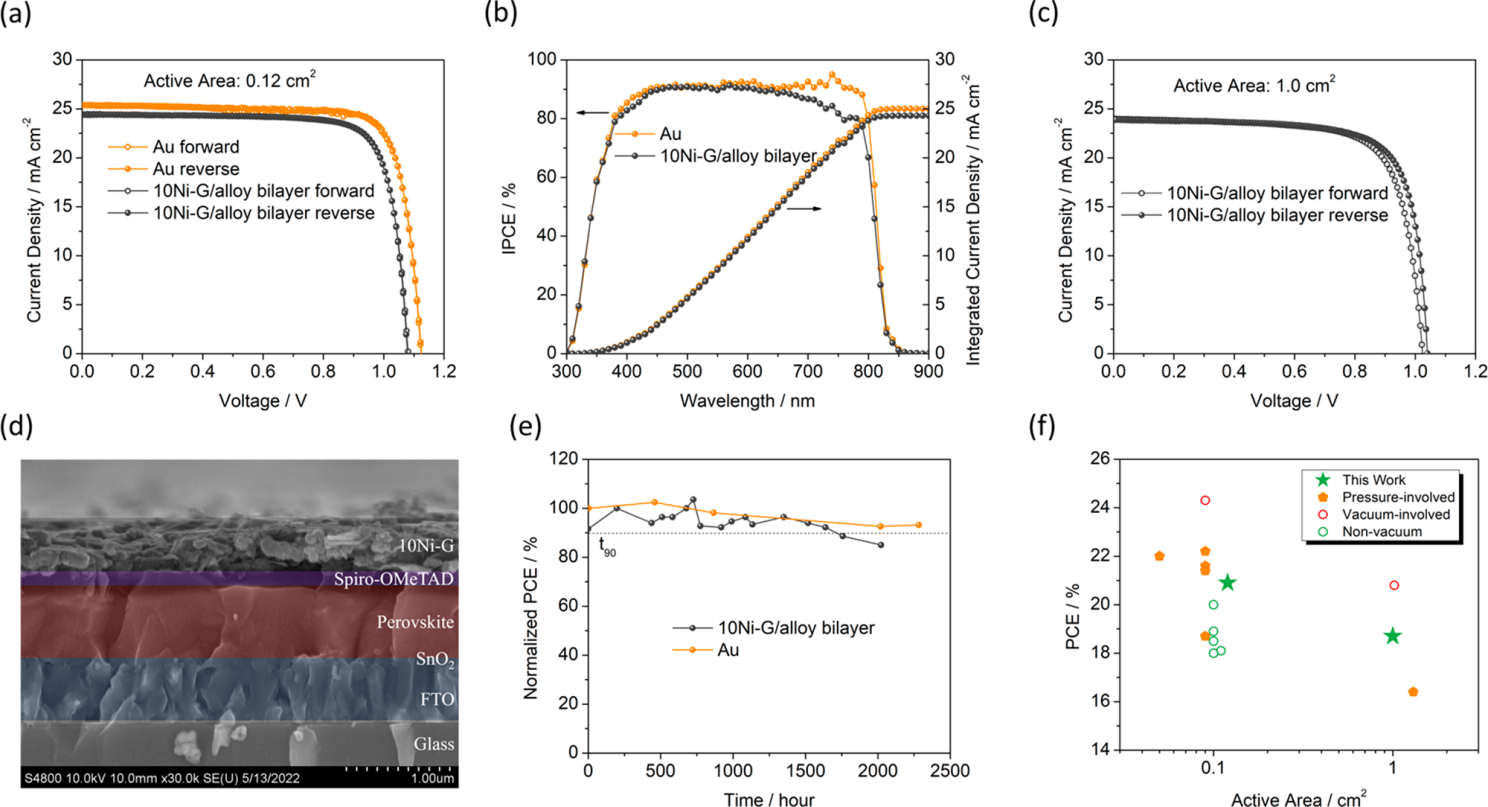
(a) *J*–*V* curves of PSC devices based on 10Ni-G/alloy and Au back electrodes. (b) IPCE spectra and integrated current density of Au-based and 10Ni-G/alloy-based PSC devices. (c) *J*–*V* curves of a 10Ni-G/alloy-based PSC device with a large active area (1 cm^2^). (d) Cross-section SEM image of a 10Ni-G/alloy-based PSC device. (e) Long-term stability of Au-based and 10Ni-G/alloy-based PSC devices under ambient storage conditions. (f) Statistical distribution of reported PCEs and active areas of PSCs with different types of back electrodes, including those processed by vacuum deposition and those packed under pressure.

**Table 2:** PV Parameters (Reverse Scan) of PSC Devices Based on 10Ni-G/Alloy with a Small Active Area (0.12 cm^2^), 10Ni-G/alloy with a Large Active Area (1 cm^2^), and Au with a Small Active Area (0.12 cm^2^)

back electrode	area (cm^2^)	*J*_sc_ (mA cm^–2^)	*V*_oc_ (V)	FF	PCE (%)
10Ni-G/alloy bilayer	0.12	24.4	1.08	0.79	21.0
10Ni-G/alloy bilayer	1.0	23.9	1.04	0.75	18.7
Au	0.12	25.4	1.13	0.80	22.8

Figure 3d shows the cross-sectional SEM image of the FAPbI_3_-based PSC using 10Ni-G as the back electrode. Note that the alloy layer is not presented, as it is too ductile to be broken with a sharp edge for SEM study. Figure 3e compares the ambient storage stability of PSCs based on 10Ni-G bilayer and Au back electrodes, with an average relative humidity in the range of ∼40–50%. Note that the Au-based PSC was encapsulated to prevent moisture uptake. In contrast, the 10Ni-G-based PSC was not encapsulated, as the 10Ni-G bilayer is so dense that it provides an effective encapsulation for mitigating moisture ingress. The 10Ni-G device maintained 85% of its initial efficiency (*T*_85_) for more than 2000 h, only slightly shorter than the *T*_90_ = 2282 h for the gold-based encapsulated device. After 196 h of thermal aging of 70 °C in an ambient atmosphere, a PCE device based on a 10Ni-G/alloy bilayer electrode maintained 86% of its initial PCE, while an Au-based device exhibited 77% of its initial PCE (Figure S14). Operational stability tracking at the maximum power point (Figure S15) shows that the unencapsulated 10Ni-G device maintained ∼86% of its initial PCE after 500 h operation under AM 1.5G illumination (100 mW/cm^2^) in an ambient atmosphere (about 10–20% relative humidity). Figure 3f plots the PCE against the device active area for our PSCs and other reported PSCs, all of which use a carbon-based back electrode. When compared with vacuum-free processed PSCs without pressurization, our work stands out in both PCE and active area.

To investigate the economic feasibility for large-scale industrial applications, the costs of various back electrodes are calculated based on the reported manufacturing techniques (Tables S1 and S2). A brief technoeconomic analysis of the cost of raw materials suggests that for a PSC-based solar plant with 1 gigawatt (GW) of power output, our 10Ni-G/alloy back electrode will result in a factor of ∼4–1000 cost reduction compared to other types of back electrode materials, as shown in Table S2. In addition to the markedly reduced materials cost, we also compared the manufacturing complexity and cost of our nickel-doped natural graphite with those of the nanostructured carbon materials used by others as components in PSC back electrodes (Table S3). Our 10Ni-G/alloy bilayer back electrode uses only low-cost and simple tools that can be readily scaled up, whereas other nanostructured carbons all involve the use of capital-intensive manufacturing facilities and equipment.

In summary, we present an innovative bilayer structured back electrode composed of a layer of low-cost Ni-doped natural graphite for interfacial charge extraction and a fusible metal alloy layer for charge transport. In addition to circumventing the use of costly gold as the back electrode, this method can be readily implemented under ambient conditions without involving any costly vacuum deposition processes or complex pressurization fixtures in the final devices. Thus, this disruptive method promises a very significant reduction in materials cost and infrastructure investment to accelerate the industrialization and commercialization of PSCs.

## Supporting Information Available

Materials, material synthesis and preparation method, device fabrication protocol, characterizations, synthetic diagram, photos, SEM images, XRD spectra, and EDX mapping of Ni-doped graphite, phase diagram of Bi-In alloy, photos of rubbing graphite and brushing alloy, cross-section diagram and SEM image of PSC device, long-term storage stability of PSC based on Au and 10Ni-G, statistical summary and comparison of PCE, area, cost evaluation, and fabrication process according to reported works (PDF)
