# Supplementary material for: Nickel-Doped Graphite and Fusible Alloy Bilayer Back Electrode for Vacuum-Free Perovskite Solar Cells
Source: ACS Energy Lett. 2023 Jun 7;8(7):2940–5. doi: 10.1021/acsenergylett.3c00852 (PMC10353479; doi:10.1021/acsenergylett.3c00852)
Supplement: Supplementary file 1 — nz3c00852_si_001.pdf [file nz3c00852_si_001.pdf]

SUPPORTING INFORMATION

Nickel-Doped Graphite and Fusible Alloy Bilayer Back Electrode for Vacuum-Free Perovskite Solar Cells

Mengyuan Li,[1#] *So Yeon Park,*[2#] *Jianxin Wang,*[1] *Ding Zheng,*[3] *Owen S. Wostoupal,*[1] *Xudong Xiao,*[1] *Zhenzhen Yang,*[4] *Xun Li,*[1] Benjamin T. Diroll,[5] *Tobin J. Marks,*[3] *Kai Zhu,*[2*] *Tao Xu*[1*]

[1] Department of Chemistry and Biochemistry, Northern Illinois University, DeKalb, Illinois 60115, United States

[2] Chemistry and Nanoscience Center, National Renewable Energy Laboratory, Golden, Colorado 80401, United States

[3] Department of Chemistry and the Materials Research Center, Northwestern University, Evanston, Illinois 60208, United States

[4] Chemical Sciences and Engineering Division, Argonne National Laboratory, Lemont, Illinois 60439, United States

[5] Center for Nanoscale Materials, Argonne National Laboratory, Lemont, Illinois 60439, Illinois 60439, United States

* Corresponding Authors: kai.zhu@nrel.gov (K.Z.); txu@niu.edu (T.X.)

[#] M.L. and S.Y.P. contributed equally to this work.

Table of Content

1. Materials S4

2. Material Synthesis and Preparation S4

3. Device Fabrication....................S5

4. Characterization....................S8

Figure S1....................S10

Figure S2....................S11

Figure S3....................S12

Figure S4....................S13

Figure S5....................S14

Figure S6....................S15

Figure S7....................S16

Figure S8....................S17

Figure S9....................S18

Figure S10....................S19

Figure S11....................S20

Figure S12....................S21

Figure S13....................S22

Figure S14....................S23

Figure S15........S24

Table S1. ....S25

Table S2. ....S26

Table S3. ....S27

References........S28

1. Materials

All chemicals used in this work are commercially available, except where indicated otherwise. *N*,*N*-dimethylformamide (DMF), dimethyl sulfoxide (DMSO), chlorobenzene (CB), tin(Ⅱ) chloride dihydrate ($SnCl_2·2H_2O$, 99.99%), urea, thioglycolic acid (TGA), hydrochloric acid (HCl, 37%), 4-tert-butylpyridine (*t*BP), acetonitrile (ACN), methylammonium bromide (MABr), 2,2',7,7'-Tetrakis(*N*,*N*-dip-methoxylphenylamine)-9,9'-spirobifluorene (spiro-OMeTAD, SHT-263), nickel dichloride hexahydrate ($NiCl_2·6H_2O$), sodium borohydride ($NaBH_4$), and FK209 Co(Ⅲ) TFSI salt were purchased from Sigma-Aldrich. Bis(trifluoromethane)sulfonimide lithium salt (Li-TFSI), cesium iodide (CsI), lead iodide (PbI_2), and lead bromide ($PbBr_2$) were purchased from TCI America. Formamidinium iodide (FAI) was purchased from Greatcell Solar. FTO (TEC 7) glass was purchased from NSG Pilkington. Natural graphite flake (325 mesh, 99.8%) was purchased from Alfa Aesar. Kapton tapes, foam swabs, and paint brushes were purchased from Amazon.

2. Material Synthesis and Preparation

Synthesis of nickel nanoparticles:

Exactly 200 mL of ultrapure water was added to a beaker with 5.0 g $NiCl_2·6H_2O$ and stirred to obtain a transparent green solution. Next, 300 mg of $NaBH_4$ was carefully added into the $NiCl_2$ solution in batches. Black nickel particles immediately precipitated with vigorous effervescence. Stirring was continued for 5 min. Then, another 300 mg of $NaBH_4$ was added into the solution and stirred for another 5 min. For the complete reduction of Ni^{2+} to Ni^0, another 400 mg of $NaBH_4$ was added into the solution under stirring for at least 30 min. The precipitated black nickel particles were collected by filtration, and at least total 400 mL of ultrapure water was used to rinse nickel particles 10 times. Finally, the collected Ni particles were put on the hot plate for desiccation at 110°C for 2 h.

Preparation of 10 wt% nickel-doped graphite:

Exactly 10 mg of dried Ni nanoparticles and 100 mg of natural graphite flake were added to a 20-mL vial with 6 mL of anhydrous ethanol. Ultrasonic treatment for 30 min was employed for dispersion and mixing. The suspension was then poured into a weighing bowl and put on the hot plate for desiccation at 110°C for 1 h.

Preparation of bismuth-indium (Bi-In) alloy:

Bismuth and indium metals of exactly the same weight were placed in the Ni crucible. The crucible was heated at 200°C for the In to melt. A wood stick was used to stir the melting In to dissolve the Bi solid. When all metals formed a homogeneous liquid phase, the hot plate was turned off. The Bi-In alloy ingot was obtained after cooling down to room temperature.

3. Device Fabrication

FTO substrates were chemically etched with zinc power and dilute HCl solution. Then, the etched FTO substrates were ultrasonically cleaned with a detergent solution made from Hellmanex detergent concentrate and deionized water. Next, deionized water was used to repeatedly rinse the FTO glass to remove residual detergent solution. The FTO glass was dried with a hot air gun. Kapton tape was stuck on the counter electrode part of the FTO to avoid further SnO_2 deposition. All FTO substrates were arranged vertically on a customized Polytetrafluoroethylene (PTFE) holder, and the holder was placed in a glass container. The PTFE holder and glass container must be ultrasonically cleaned by a concentrated KOH aqueous solution in advance to remove all the precipitated contaminants from previous SnO_2 deposition reactions. These contaminants would severely affect subsequent SnO_2 depositions and cause poor device performance. For the SnO_2 deposition, the chemical bath deposition (CBD) method[1] was slightly modified. Thus, 6.25 g of urea was added into a glass bottle with 500 mL of ultrapure water. After the solution was stirred to be entirely clear, 1.36 g of $SnCl_2 \cdot 2H_2O$ was added into the glass bottle. The solution initially

became turbid because $SnCl_2 \cdot 2H_2O$ hydrolyzed in the alkaline aqueous solution. After the solution was stirred and became stable semitransparent colloidal solution (about 2 min), 6.25 mL of 37% concentrated HCl was added to the colloidal solution to dissolve all the precipitate. Finally, 125 μL of TGA was added to this transparent solution. Stirring was continued for about 2 min, and then the solution was poured into the glass container with the FTO substrates. A hot plate was used to heat the glass container at 160°C for 4 h to achieve CBD SnO_2 ETL deposition. The temperature of the precursor solution was over 90°C. After the reaction was complete, the SnO_2-deposited FTO substrates were ultrasonically cleaned by repeated deionized water treatment. Water on the deposited FTO glass was blown away by a hot air gun. The deposited FTO substrates were then placed on the hot plate to anneal for 1 h at 170°C.

To fabricate the perovskite active layer with the composition of $(FA_{0.85}MA_{0.1}Cs_{0.05})Pb(I_{0.9}Br_{0.1})_3$, we prepared a perovskite precursor solution by dissolving FAI (205 mg, 1.19 M), MABr (16 mg, 0.14 M), CsI (18 mg, 0.07 M), $PbBr_2$ (51 mg, 0.14 M), and PbI_2 (599 mg, 1.3 M) in the solvent, a mixture of 800 μL of DMF and 240 μL of DMSO. Then, 30 μL of perovskite precursor solution was dripped onto the SnO_2 substrate. The perovskite layer was deposited by a two-step spin coating procedure at 2,000 rpm (ramp: 200 rpm/s) and 6,000 rpm (ramp: 2,000 rpm/s) for 10 s and 20 s, respectively. At the last 6 s of the second step, 120 μL of chlorobenzene (CB) was dripped onto the perovskite surface within 1.5 s. The perovskite layer gradually turned brown after CB dripping. The substrate was immediately put on a hot plate to anneal at 120°C for 20 min. To prepare the $FAPbI_3$-based perovskite precursor solution with 3% $MAPbBr_3$ additive, 1.6 M $FAPbI_3$ perovskite precursor was prepared by mixing 1.6 M FAI and 1.6 M PbI_2 with 3 mol% $MAPbBr_3$ and 35 mol% MACl in a mixed solution of DMF and DMSO (8:1 v/v). Then the perovskite precursor solution was deposited onto the UV-ozone treated SnO2 film at 5000 rpm for 20s, where 1 mL of diethyl ether was dropped on the rotating film 10 s after spinning. The resulting film was annealed at 150 °C for 15 min and 100 °C for 5 min, sequentially. To prepare the spiro-OMeTAD precursor solution, 86 mg of spiro-OMeTAD was dissolved in 1 mL of CB. Then, 34.15 μL of *t*BP, 19.44 μL of Li-TFSI ACN solution (1.8 M, 520 mg in 1 mL ACN), and 10.5 μL of FK209 Co(Ⅲ) TFSI

ACN solution (0.2 M, 300 mg in 1 mL ACN) were added into the spiro-OMeTAD CB solution accordingly. Next, 30 μL of spiro-OMeTAD precursor solution was dripped onto the perovskite layer; the spiro-OMeTAD layer was deposited by a spin coating procedure at 4,000 rpm (ramp: 2,000 rpm/s) for 20 s. Before coating the bilayer back electrode, the substrates were placed in dry ambient environment for at least 24 h for the oxidation of the spiro-OMeTAD layer with the oxygen in air.

Before coating the bilayer back electrode, we stuck Kapton tape onto the edges of the spiro-OMeTAD film to avoid a short circuit. Then, Kapton tape of 3-mm width was stuck precisely down the middle of the whole area, dividing the surface into two separate areas. In this way, two independent subcells were obtained on one device. Photos of the substrates with a scaled ruler for reference were taken to calculate the active area (the area was calculated by measuring the pixel and comparing the pixel area with the scale of the ruler). For the fixed aperture area of 0.1 cm^2, a shadow mask with two separate aperture areas was attached the glass side of the device. We fully stained foam swabs with graphite powders or Ni-doped graphite powders. Then, the powders were painted onto the spiro-OMeTAD surface—lightly at first, and then more strongly—until the compact surface was observed. Note that the graphite layers of the two separate areas should not cross over to each other. A small piece of glass can be used to cover one area when painting graphite powder onto another area. After fabricating the Ni-doped graphite, we stained a foam swab with pure graphite and painted it onto the Ni-doped graphite because Ni does not have an affinity for the Bi-In alloy. It is necessary to coat pure graphite before brushing the Bi-In alloy because pure graphite can greatly improve the wettability of the Bi-In alloy. Finally, the graphite-coated devices were placed on the hot plate at 110°C. A paintbrush was fully stained with molten Bi-In alloy, which was then brushed onto the graphite surface. After brushing, the device was continuously heated for about 1 min and then placed on a cool plate for the alloy to solidify. Similarly, the alloy layers of the two separate areas should not cross over to each other. Polydimethylsiloxane (PDMS) silicone elastomer (Sylgard 184) and its corresponding curing agent were used in a volume ratio of 10:1 to encapsulate the devices.

4. Characterization

X-ray photoelectron spectroscopy (XPS) was conducted in a PHI 5000 VersaProbe II system (Physical Electronics) attached to an Ar-atmosphere glovebox. The spectra were obtained using an Al Kα radiation (hυ = 1486.6 eV) beam (100 μm, 25 W), with Ar+ and electron beam sample neutralization, in a fixed analyzer transmission mode. Ultraviolet photoelectron spectroscopy (UPS) measurements were performed on a Thermo Scientific ESCALAB 250Xi photoelectron spectrometer at a base pressure of 2×10^{-8} mbar. The UPS used a He-I light (21.2182 eV) as the UV source. X-ray diffraction (XRD) measurements were performed by a Bruker D8 Advance A25 X-ray diffractometer. Scanning electron microscopy (SEM) and energy-dispersive X-ray spectroscopy (EDX) measurements were performed by a Jeol 7500F field emission scanning electron microscope and a Tescan Vega II scanning electron microscope, respectively. Resistivity measurements were performed by a Jandel four-point sheet resistance measurement system. The current-voltage (*J-V*) characteristic curves were obtained by a Keithley 2401 source meter under AM 1.5G illumination (100 mW/cm^2) provided by a Newport Oriel Sol3A solar simulator. The *J-V* curves were measured with voltage scan from −0.2 V to 1.2 V at a scanning rate of 50 mV/s. Incident photon to current conversion efficiency (IPCE) measurements were performed by a QEXL solar cell quantum efficiency measurement system. Steady-state photoluminescence (SSPL) and time-resolved photoluminescence (TRPL) measurements were performed by 400-nm laser from a diode laser or light source within a streak camera system (Hamamatsu C5680).

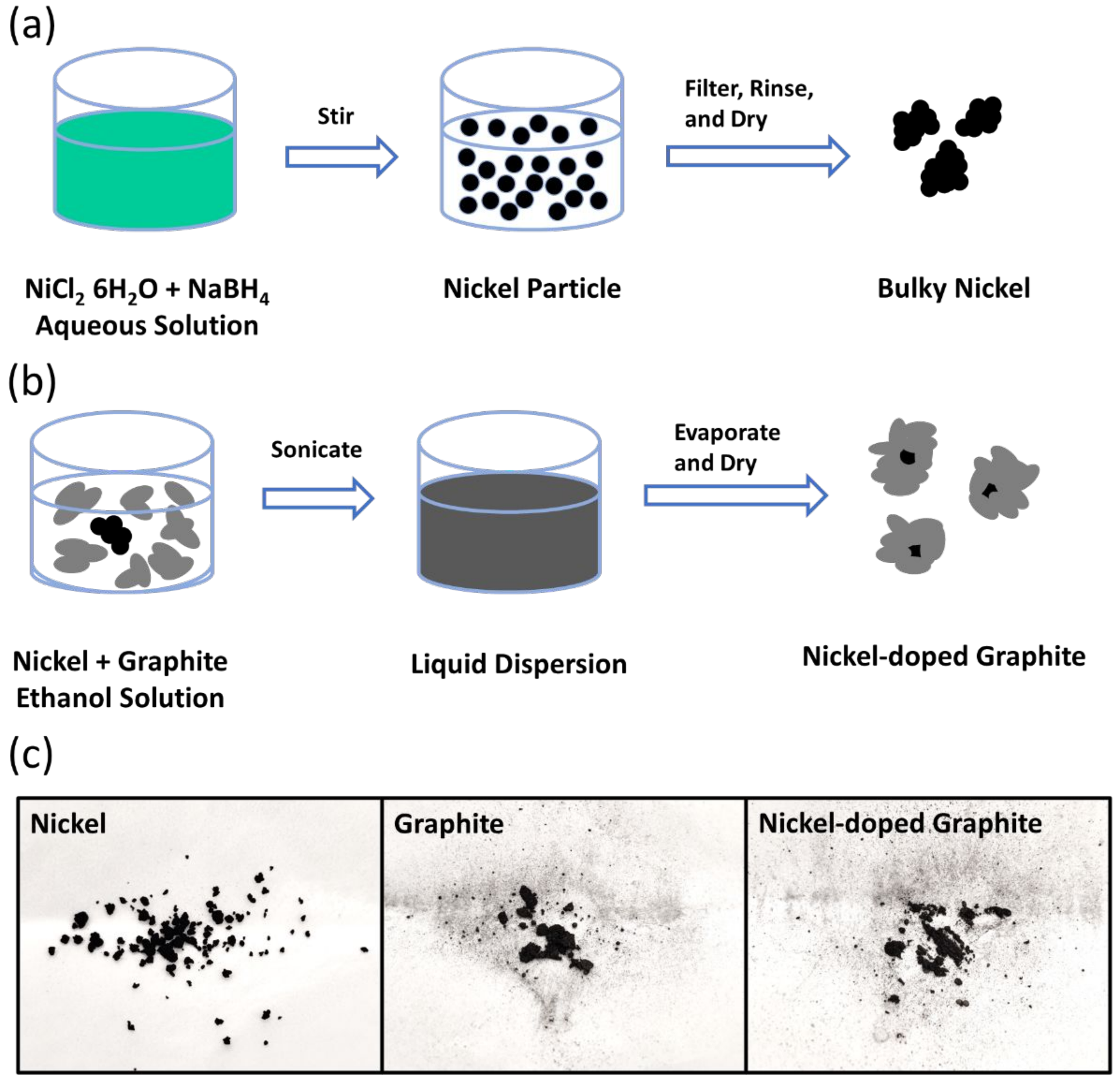

Figure S1. (a) Schematics illustrating the synthesis of bulky nickel particles. (b) Schematics illustrating the synthesis nickel-doped graphite. (c) Photos of bulky nickel particles, natural graphite powder, and nickel-doped graphite powder.

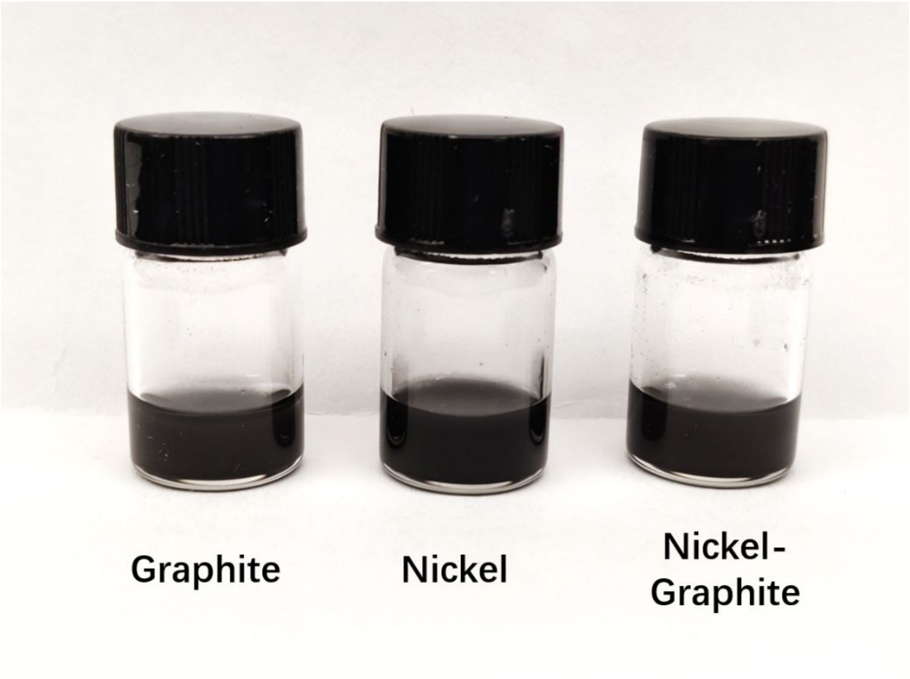

Figure S2. Photo of the ethanol dispersion solutions of graphite, nickel, and nickel-doped graphite.

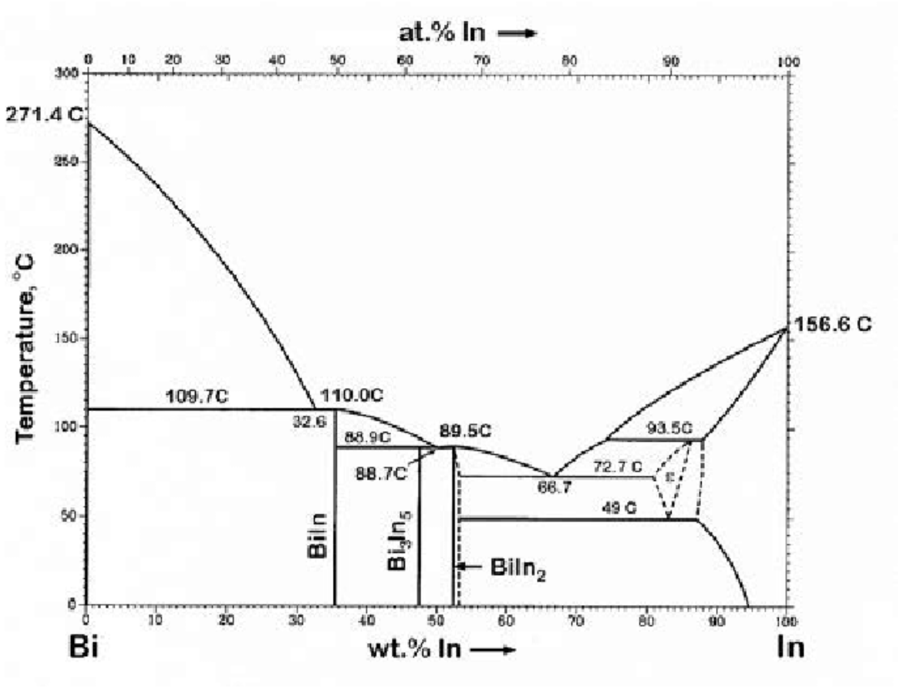

Figure S3. Weight ratio dependent phase digram of bismuth and indium.[2]

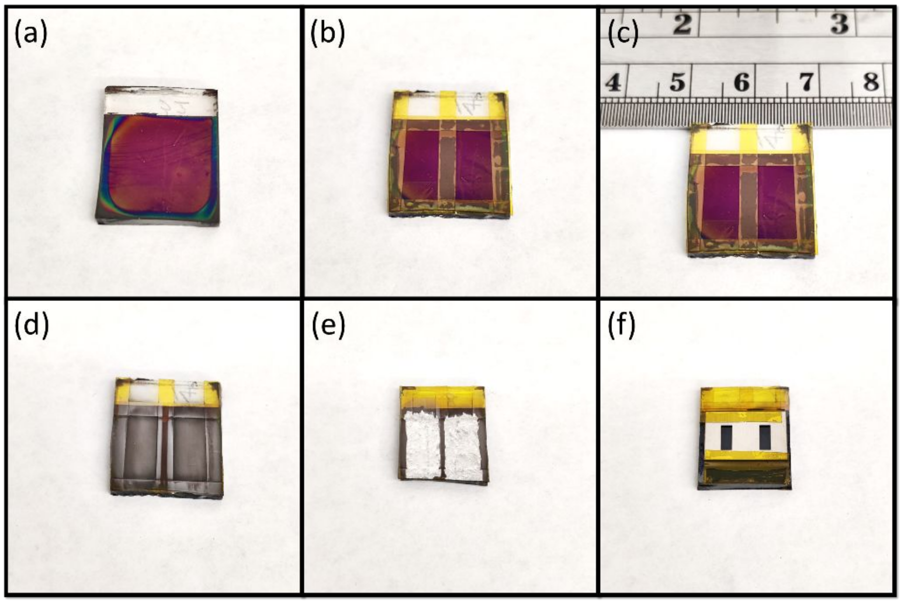

Figure S4. (a) Uncoated spiro-OMeTAD substrate. (b) Kapton tape was stuck on the substrate to divide the whole area into two independent parts. (c) A scale ruler was used as a reference to calculate the active area. (d) Spiro-OMeTAD substrate coated by Ni-doped graphite. (e) Ni-doped graphite substrated coated by Bi-In alloy. (f) Customized shadow mask was stuck on the glass side of the device.

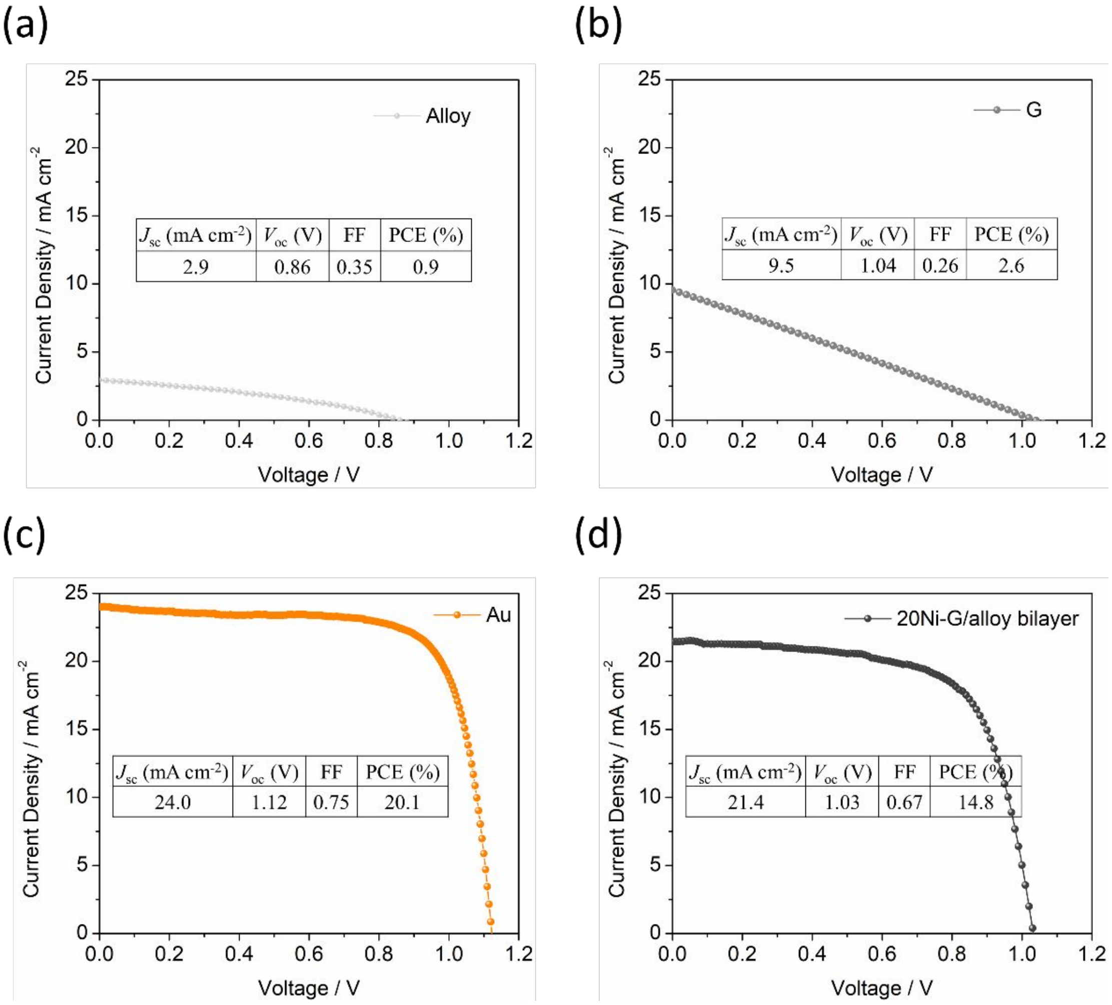

Figure S5. *J*-*V* curves of perovskite solar cell devices based on alloy (a), G (b), Au (c), and 20Ni-G (d) back electrodes. Inset table shows J_{sc}, V_{oc}, FF and PCE for each kind of devices.

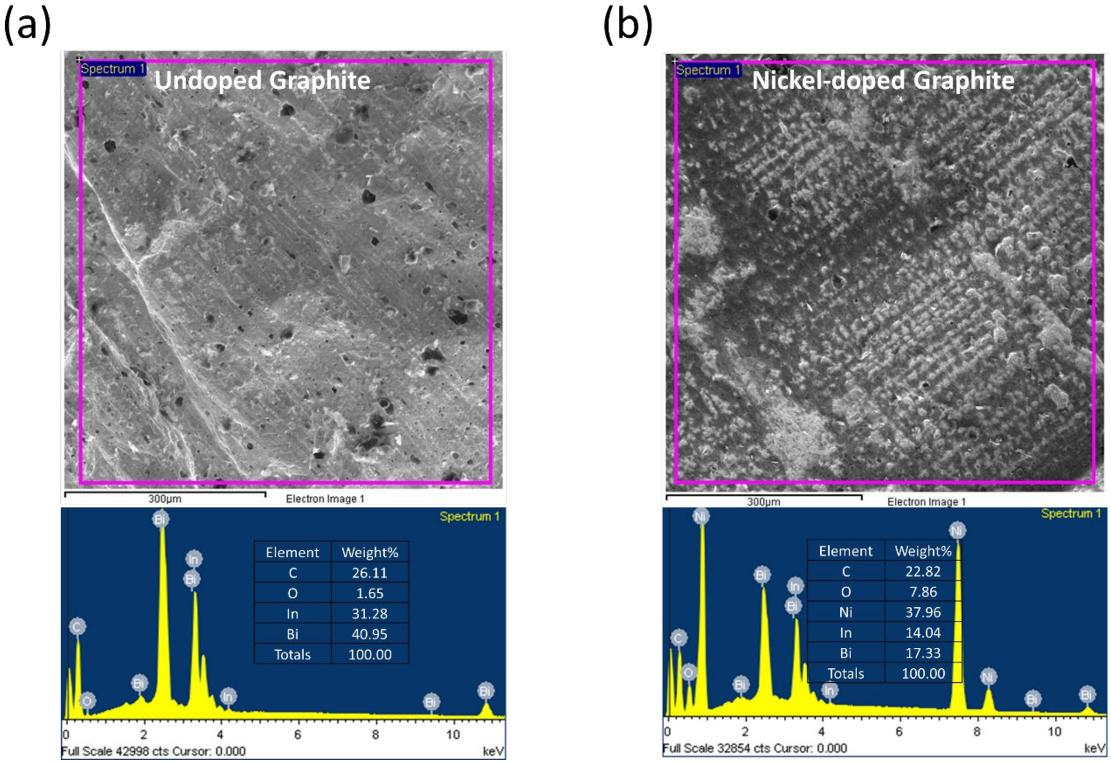

Figure S6. EDX element analysis of undoped graphite/Bi-In alloy and Ni-doped graphite/Bi-In alloy.

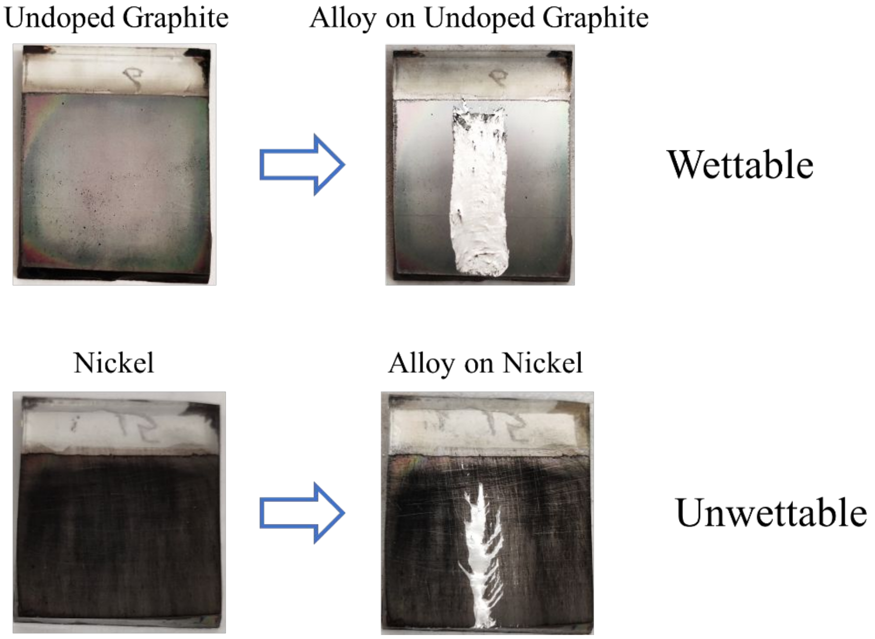

Figure S7. Photos to show the wettability of Bi-In alloy on undoped graphite and on nickel. It is clear that the Bi-In alloy showed very good wettability on the pure graphite layer but poor wettability on the Ni microparticle layer.

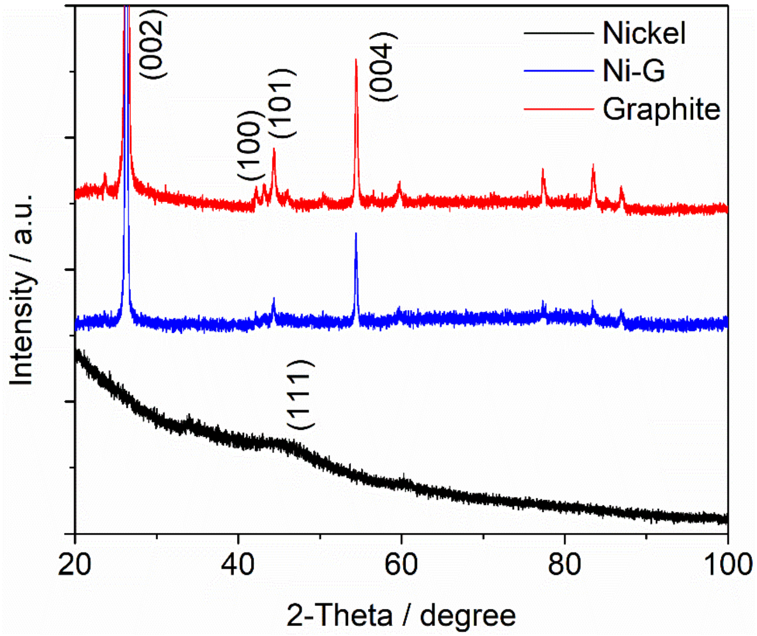

Figure S8. XRD spectra of graphite, nickel, and nickel-doped graphite. The absence of any diffraction patterns in the nickel microparticles clearly shows the amorphous nature of the Ni microparticles. The characteristic XRD peaks for graphite are clearly identified.

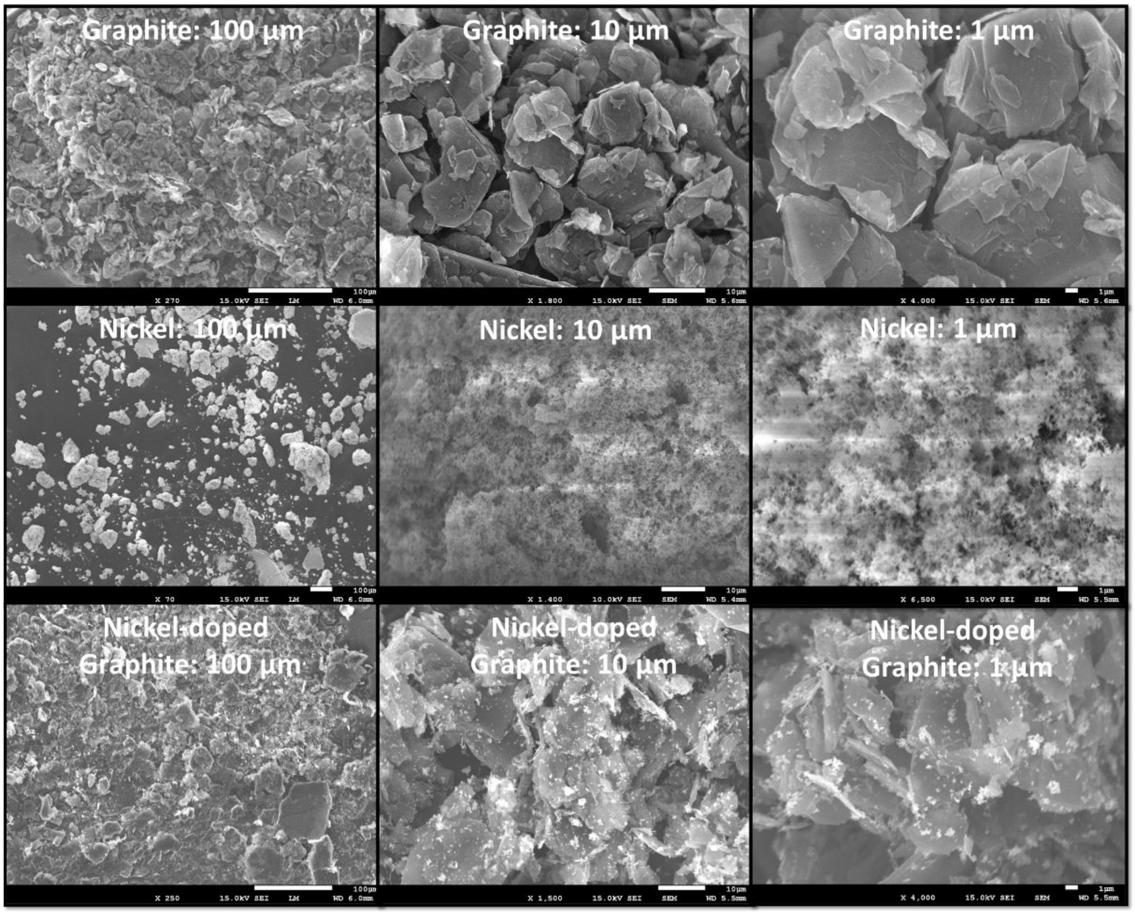

Figure S9. SEM images with 100-μm, 10-μm, and 1-μm scale bars for natural graphite, nickel particles, and nickel-doped graphite, respectively.

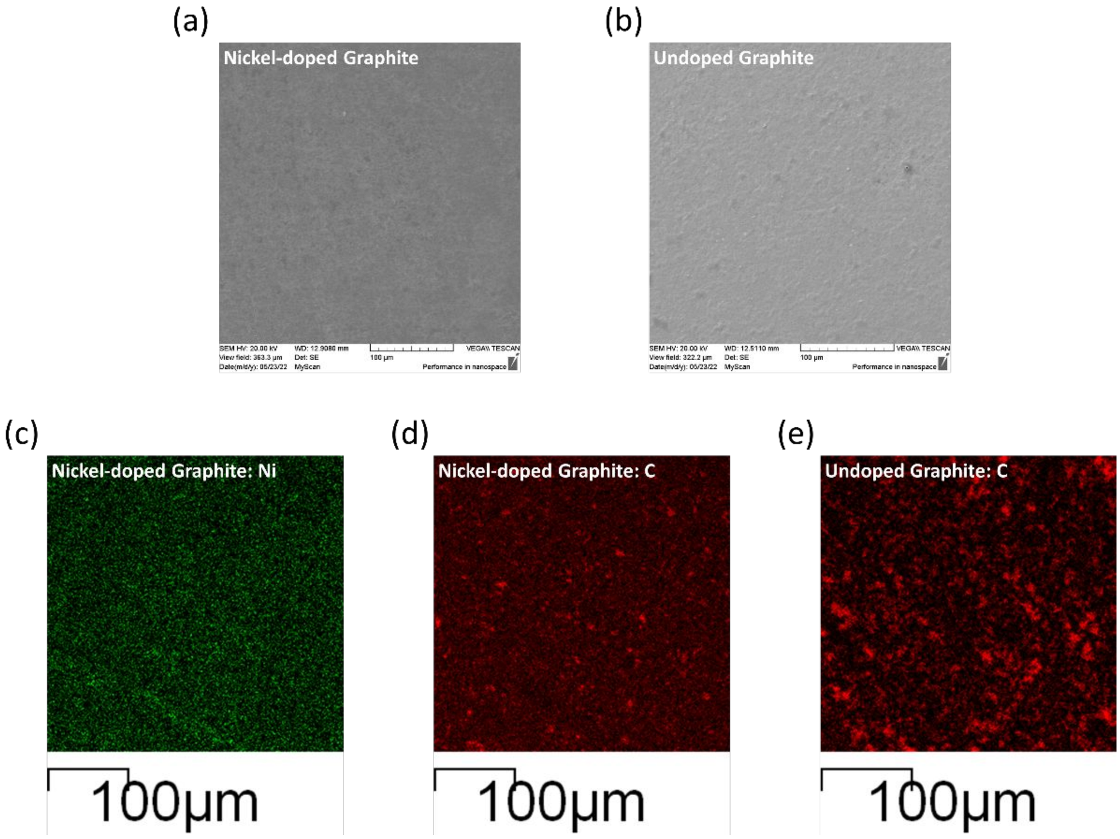

Figure S10. Surface SEM images of nickel-doped graphite (a) and undoped graphite (b). Surface EDX mapping of Ni element (c) and C element (d) for the surface of nickel-doped graphite, and C element (e) for the surface of undoped graphite.

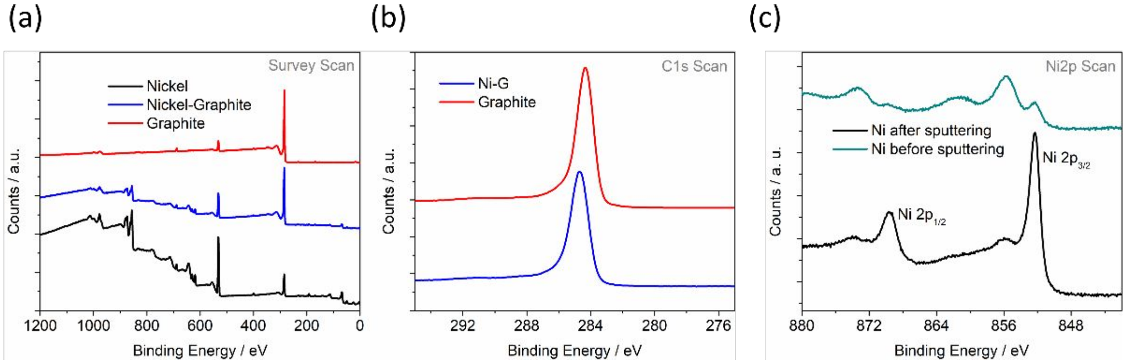

Figure S11. (a) XPS survey scan spectra of graphite, nickel, and nickel-doped graphite. (b) XPS C1s scan spectra of graphite and nickel-doped graphite. (c) XPS Ni2p scan spectra of nickel and nickel-doped graphite. After removal of the inevitable surface nickel oxide layer (binding energy = 855.7 eV for Ni^{2+}) by Ar^{+} sputtering, the binding energy of the Ni metal particle is 852.2 eV, in good agreement with that of metallic Ni in the literature.[3]

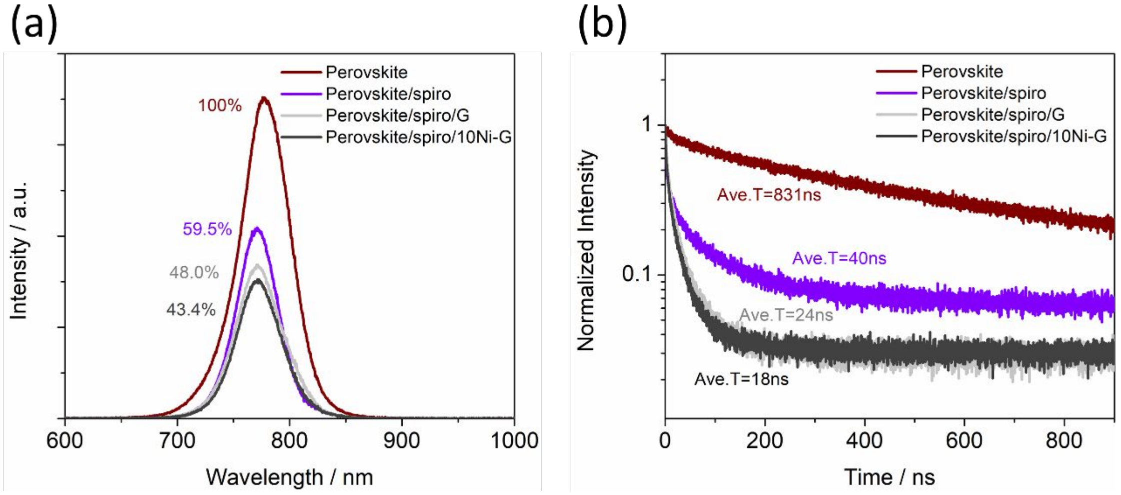

Figure S12. Steady-state photoluminescence spectra (a) and time-resolved photoluminescence spectra (b) of samples with structures of glass/perovskite, glass/perovskite/spiro-OMeTAD, glass/perovskite/spiro-OMeTAD/G, glass/perovskite/spiro-OMeTAD/10Ni-G, respectively.

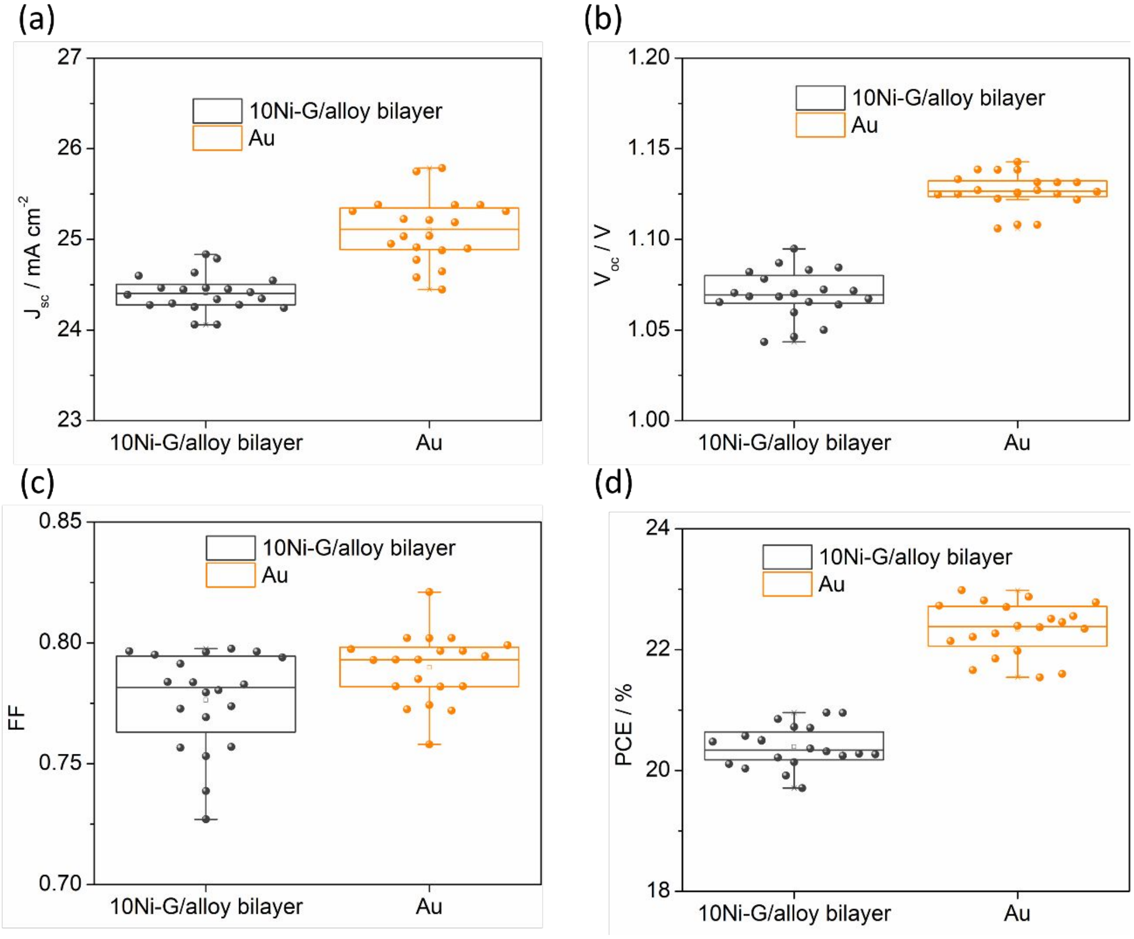

Figure S13. Statistical PV parameter boxplots of PSC devices based on 10Ni-G/alloy and Au back electrodes.

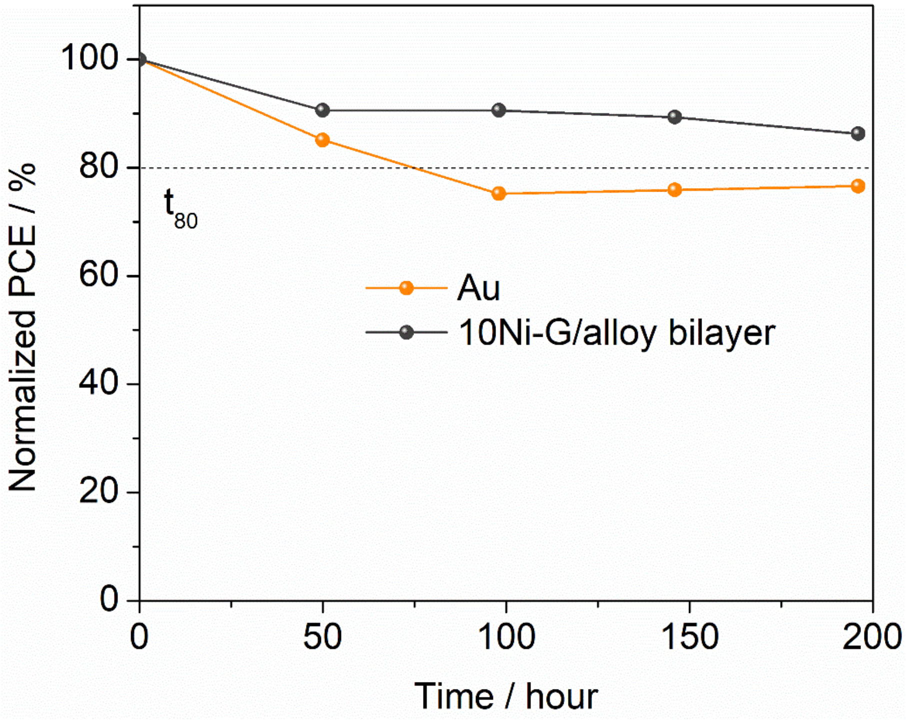

Figure S14. Thermal stability of Au-based and 10Ni-G/alloy-based PSC devices under 70°C heating in ambient atmosphere.

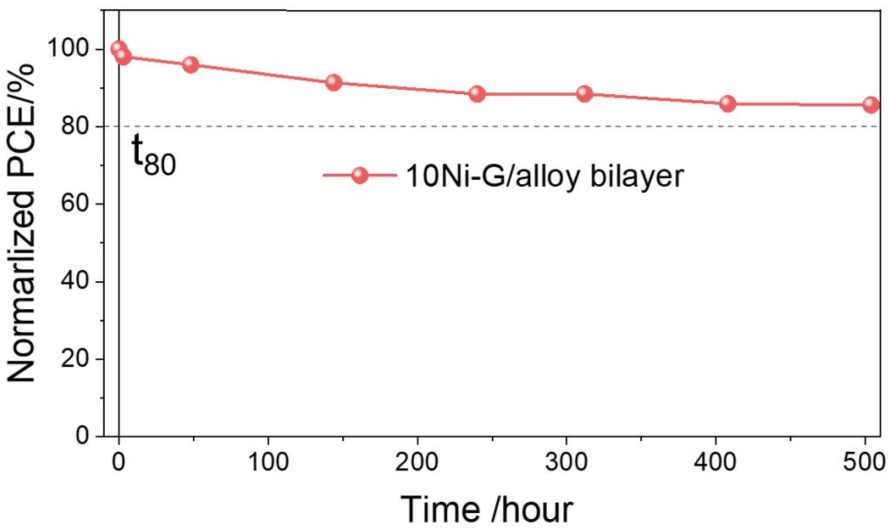

Figure S15. Operational stability at maximum power point of 10Ni-G/alloy-based PSC device under AM 1.5G illumination (100 mW/cm^2) at 30°C in ambient atmosphere with 10~30% relative humidity.

Table S1. Abundance and price of the materials used for the back electrode.

**Materials**	**Abundance or Reserve in Earth's Crust**	**Price per Gram**
Gold	0.004 ppm	60 USD
Bismuth	0.85 ppm	0.1 USD
Indium	2.4 ppm	0.9 USD
Natural graphite	800 million tons	0.2 USD

Table S2. Manufacturing cost evaluation of various back electrodes, according to the reported works.

Back Electrode Structure	Raw Materials and Commercial Price	Usage of Materials for 1 cm^2 Coating Area	Electrode Processing Technique	Active Area	PCE	Cost Evaluation of Large-scale Industrialization at 1 GW Solar Panels	Reference
Mesoporous Carbon	Carbon Paste: 0.2 USD/gram	Carbon Paste: 0.1 gram	Screen Coating; 400 °C Sintering	0.108 cm^2	18.1%	0.11 billion USD	*J. Phys. Chem. Lett.* **2022**, *13*, 2144–2149.
Carbon Film	Carbon Paste: 0.2 USD/gram	Carbon Paste: 0.2 gram	Doctor Blade Coating; Solvent Exchange Mechanical Pressing	0.1 cm^2	20.0%	0.21 billion USD	*Adv. Funct. Mater.* **2018**, *28*, 1802985.
Few-layer Graphene / Al Foil	Graphene: 150 USD/gram Aluminum: 0.06 USD/gram	Graphene: 0.01 gram Al: 0.06 gram	Spray Coating; Assembling under Pressure	0.09 cm^2	18.7%	8.02 billion USD	*Energy Environ. Sci.* **2019**, *12*, 3585-3594.
Few-layer Graphene / FTO	Graphene: 150 USD/gram FTO: 0.015 USD/gram	Graphene: 0.01 gram FTO: 1 cm^2	Spray Coating; Assembling under Pressure	1.3 cm^2	16.4%	9.24 billion USD	*Energy Environ. Sci.* **2019**, *12*, 3585-3594.
SA-Ti rGO / FTO	rGO: 300 USD/gram FTO: 0.015 USD/cm^2	SA-Ti rGO: 0.02 gram: FTO: 1 cm^2	Spray Coating; Assembling under Pressure	0.09 cm^2	21.6%	27.85 billion USD	*Nat. Energy* **2021**, *6*, 1154–1163.
SWCNT /FTO	SWCNT: 500 USD/gram FTO: 0.015 USD/cm^2	SWCNT: 0.01 gram FTO: 1 cm^2	Spray Coating; Assembling under Pressure	0.09 cm^2	21.4%	23.43 billion USD	*Energy Environ. Sci.* **2022**, *15*, 1536-1544.
MWCNT / FTO	MWCNT: 10 USD/gram FTO: 0.015 USD/cm^2	MWCNT: 0.01 gram FTO: 1 cm^2	Spray Coating; Assembling under Pressure	0.09 cm^2	22.2%	0.58 billion USD	*Energy Environ. Sci.* **2022**, *15*, 1536-1544.
MWCNT / FTO	MWCNT: 10 USD/gram FTO: 0.015 USD/cm^2	MWCNT: 0.01 gram FTO: 1 cm^2	Spray Coating; Assembling under Pressure	0.049 cm^2	22.0%	0.58 billion USD	*Adv. Funct. Mater.* **2022**, *32*, 2204831.
Graphene-doped EVA / Ni-Cu-Graphene	Monolayer Graphene: 3 USD/cm^2 Ni-coated Copper Foil: 0.02 USD/cm^2	Monolayer Graphene: 1 cm^2 Ni-coated Copper Foil: 1 cm^2	Magnetron Sputtering; Chemical Vapor Deposition; Hot Pressing	0.09 cm^2	24.3%	12.43 billion USD	*Nat. Energy* **2022**, *7*, 520–527.
Graphene-doped EVA / Ni-Cu-Graphene	Monolayer Graphene: 3 USD/cm^2 Ni-coated Copper Foil: 0.02 USD/cm^2	Monolayer Graphene: 1 cm^2 Ni-coated Copper Foil: 1 cm^2	Magnetron Sputtering; Chemical Vapor Deposition; Hot Pressing	1.02 cm^2	20.8%	14.52 billion USD	*Nat. Energy* **2022**, *7*, 520–527.
10% Ni-doped Graphite / Bi-In Alloy	10% Ni-doped Graphite: 0.1 USD/gram Bi-In Alloy: 0.25 USD/gram	10% Ni-doped Graphite: 0.01 gram Bi-In Alloy:0.02 gram	Mechanical Rubbing; 110 °C Brushing	0.12 cm^2	20.9%	0.03 billion USD	This Work
10% Ni-doped Graphite / Bi-In Alloy	10% Ni-doped Graphite: 0.1 USD/gram Bi-In Alloy: 0.25 USD/gram	10% Ni-doped Graphite: 0.01 gram Bi-In Alloy:0.02 gram	Mechanical Rubbing; 110 °C Brushing	1.0 cm^2	18.7%	0.04 billion USD	This Work

Table S3. Fabrication techniques of various back electrodes, according to the reported works.

**Carbon Materials**	**Synthesis Method for the Relevant Carbon Materials**	**Main Equipment and Facilities for Synthesis**	**Reference**
Few-layer Graphene	Chemical Vapor Deposition	High-Temperature Furnace	*Energy Environ. Sci.* **2019**, *12*, 3585-3594.
SA-Ti rGO	Oxidative Exfoliation (Hummer's Method); Sonication Dispersion; Centrifugation; Calcination	Temperature-Controlled Reactor; Centrifuge; High-Temperature Furnace	*Nat. Energy* **2021**, *6*, 1154–1163.
SWCNT	Arc Discharge, Laser Vaporization, Chemical Vapor Deposition	High-Temperature Reactor	*Energy Environ. Sci.* **2022**, *15*, 1536-1544.
MWCNT	Arc Discharge, Laser Vaporization, Chemical Vapor Deposition	High-Temperature Reactor	*Adv. Funct. Mater.* **2022**, *32*, 2204831.
Monolayer Graphene	Chemical Vapor Deposition	High-Temperature Furnace	*Nat. Energy* **2022**, *7*, 520–527.
10% Ni-Doped Graphite	Wet Chemistry Synthesis; Sonication Dispersion	Hot Plate	This Work

References

(1) Saliba, M.; Correa-Baena, J.-P.; Wolff, C. M.; Stolterfoht, M. Phung, N.; Albrecht, S.; Neher, D.; Abate, A. How to Make over 20% Efficient Perovskite Solar Cells in Regular (n–i–p) and Inverted (p–i–n) Architectures. *Chem. Mater.* **2018**, *30*, 4193-4201.

(2) Biglari, Mo; Das, Aranav; Krassenburg, Ludo; Brom, J.H.G. Erik; van Veen, Nicolaas; Kodentsov, A. Low-temperature Soldering Using Ordered Alloys. *Proceedings of SMTA International* **2018**.

(3) Prieto, P.; Nistor, V.; Nouneh, K.; Oyama, M.; Mohammed A.-L.; Díaz, R. XPS Study of Silver, Nickel and Bimetallic Silver–Nickel Nanoparticles Prepared by Seed-Mediated Growth. *Appl. Surf. Sci.* **2012**, *258*, 8807-8813.
